# Artificial intelligence research in radiation oncology: a practical guide for the clinician on concepts and methods

**DOI:** 10.1093/bjro/tzae039

**Published:** 2024-11-13

**Authors:** Frank J P Hoebers, Leonard Wee, Jirapat Likitlersuang, Raymond H Mak, Danielle S Bitterman, Yanqi Huang, Andre Dekker, Hugo J W L Aerts, Benjamin H Kann

**Affiliations:** Artificial Intelligence in Medicine (AIM) Program, Mass General Brigham, Harvard Medical School, Boston, MA, MA 02115, United States; Department of Radiation Oncology, Dana-Farber Cancer Institute and Brigham and Women’s Hospital, Harvard Medical School, Boston, MA, MA 02115, United States; Department of Radiation Oncology (Maastro), GROW School for Oncology and Reproduction, Maastricht University Medical Center+, Maastricht, 6229ET, the Netherlands; Department of Radiation Oncology (Maastro), GROW School for Oncology and Reproduction, Maastricht University Medical Center+, Maastricht, 6229ET, the Netherlands; Artificial Intelligence in Medicine (AIM) Program, Mass General Brigham, Harvard Medical School, Boston, MA, MA 02115, United States; Department of Radiation Oncology, Dana-Farber Cancer Institute and Brigham and Women’s Hospital, Harvard Medical School, Boston, MA, MA 02115, United States; Artificial Intelligence in Medicine (AIM) Program, Mass General Brigham, Harvard Medical School, Boston, MA, MA 02115, United States; Department of Radiation Oncology, Dana-Farber Cancer Institute and Brigham and Women’s Hospital, Harvard Medical School, Boston, MA, MA 02115, United States; Artificial Intelligence in Medicine (AIM) Program, Mass General Brigham, Harvard Medical School, Boston, MA, MA 02115, United States; Department of Radiation Oncology, Dana-Farber Cancer Institute and Brigham and Women’s Hospital, Harvard Medical School, Boston, MA, MA 02115, United States; Department of Radiation Oncology (Maastro), GROW School for Oncology and Reproduction, Maastricht University Medical Center+, Maastricht, 6229ET, the Netherlands; Department of Radiology, Guangdong Provincial People’s Hospital (Guangdong Academy of Medical Sciences), Southern Medical University, Guangzhou, 510080, China; Department of Radiation Oncology (Maastro), GROW School for Oncology and Reproduction, Maastricht University Medical Center+, Maastricht, 6229ET, the Netherlands; Artificial Intelligence in Medicine (AIM) Program, Mass General Brigham, Harvard Medical School, Boston, MA, MA 02115, United States; Department of Radiation Oncology, Dana-Farber Cancer Institute and Brigham and Women’s Hospital, Harvard Medical School, Boston, MA, MA 02115, United States; Radiology and Nuclear Medicine, CARIM & GROW, Maastricht University, Maastricht, 6229ER, the Netherlands; Artificial Intelligence in Medicine (AIM) Program, Mass General Brigham, Harvard Medical School, Boston, MA, MA 02115, United States; Department of Radiation Oncology, Dana-Farber Cancer Institute and Brigham and Women’s Hospital, Harvard Medical School, Boston, MA, MA 02115, United States

**Keywords:** radiation oncology, artificial intelligence, machine learning, deep learning

## Abstract

The use of artificial intelligence (AI) holds great promise for radiation oncology, with many applications being reported in the literature, including some of which are already in clinical use. These are mainly in areas where AI provides benefits in efficiency (such as automatic segmentation and treatment planning). Prediction models that directly impact patient decision-making are far less mature in terms of their application in clinical practice. Part of the limited clinical uptake of these models may be explained by the need for broader knowledge, among practising clinicians within the medical community, about the processes of AI development. This lack of understanding could lead to low commitment to AI research, widespread scepticism, and low levels of trust. This attitude towards AI may be further negatively impacted by the perception that deep learning is a “black box” with inherently low transparency. Thus, there is an unmet need to train current and future clinicians in the development and application of AI in medicine. Improving clinicians’ AI-related knowledge and skills is necessary to enhance multidisciplinary collaboration between data scientists and physicians, that is, involving a clinician in the loop during AI development. Increased knowledge may also positively affect the acceptance and trust of AI. This paper describes the necessary steps involved in AI research and development, and thus identifies the possibilities, limitations, challenges, and opportunities, as seen from the perspective of a practising radiation oncologist. It offers the clinician with limited knowledge and experience in AI valuable tools to evaluate research papers related to an AI model application.

## Introduction

Artificial intelligence (AI) empowers machines to mimic human intelligence in tasks such as image recognition and language comprehension. The definition of AI^1^ does include robotics and recommender systems; however, the main focus of the present article relates to a subset of AI, which comprises computer programs that self-adapt their internal states to “learn” how to perform a hitherto unfamiliar task, a.k.a. “machine learning” (ML). Deep learning (DL) is a sub-specialization within ML that refers to a computer network architecture comprising a large number of small computing units called “artificial neurons” (see Text Box 1). Artificial intelligence’s potential in medicine has naturally attracted significant attention from clinical, societal, legal, and ethical interests.^2–5^

However, this attention clashes with challenges in clinical adoption.^6^ Many clinicians struggle to understand the processes happening under the surface of AI.^7^ For instance, the complex non-linear logic between input and output in DL is perceived by clinicians as opaque (ie, a “black box”).^7^ In spite of some impressive performance by DL thus far, this lack of understandability and transparency can foster distrust and scepticism towards AI as a whole. Some patients and clinicians fear that AI might replace doctors entirely.^8^ These human factors hinder AI integration into healthcare.

This paper aims to bridge the gap between the clinic and contemporary AI. We describe the processes involved in current research and development of AI applications for healthcare, and explore their possibilities and limitations from the perspective of a treating physician. The focus is on applications in radiation oncology (RO), particularly medical image analysis. We strongly believe that, with improved understanding of AI, clinicians will be empowered to obtain foundational knowledge, which is an essential first step towards developing the skills needed to evaluate AI research objectively and assess its potential impact on real-world clinical encounters.

Box 1. AI terminology and definitions.

Artificial intelligence (AI): AI is a broad field of computer science using automated systems, which can perform tasks that would require human intelligence. There are different subtypes of AI, with increasing complexity as described below.

Machine learning (ML): The use of algorithms to make predictions to unseen data, based on the computer’s ability to learn from observed input data, without being explicitly programmed.

Deep learning (DL): This is an advanced subfield of ML that relies on algorithms, utilizing artificial neural networks (ANNs) arranged in multiple layers to mimic the information interpretation and inference processes of the human brain. DL distinguishes itself through the utilization of numerous hidden node layers, which acquire diverse representations of data by abstracting them in various ways. DL algorithms use a large quantity of latent (or hidden or inferred) variables in its decision-making process.

Radiomics: This is the process of quantitative analysis of radiological imaging to be used for diagnostic or prognostic modelling. Two methodological distinct approaches can be distinguished: One that is based on predefined image features, which are derived from the spatial distribution of pixel or voxel intensities, shape, texture, and other characteristics. Through ML methods, associations between sets of image features and the predicted diagnosis or prognosis can be established. The second approach is through DL, which does not need predefined features, but rather learns directly from the entire image to find the optimal association between a predicted outcome and image features.

Computer vision: The use of AI that focuses on learning computers to identify and understand objects and people in images and videos. Similar to other applications of AI, computer vision aims to perform and automate tasks that replicate human capabilities, in this case with the goal to identify and classify objects.

Training: This is the process where a ML model learns from data. This involves feeding a dataset to the model and using algorithms to adjust the model’s parameters so it can accurately predict the target variable.

Tuning/validation: This is a process of evaluating the model’s performance on a separate subset of the data that were not used during training. This helps in assessing how well the model generalizes to new, unseen data.

Testing: After training and tuning, the model is evaluated on a separate test dataset to estimate its performance on unseen data.

## AI applications in RO workflow

**Figure 1.**
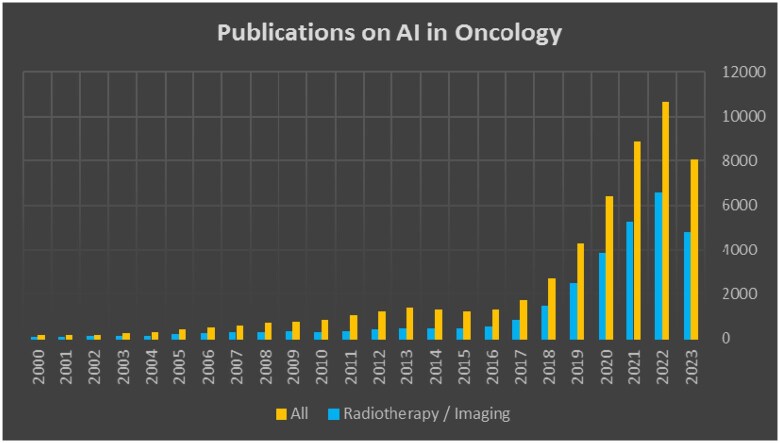
Number of annual publications on PubMed since 2000. Search query: For All: (radiomics OR deep learning OR artificial intelligence OR machine learning) AND cancer. For Radiotherapy/imaging: (radiomics OR deep learning OR artificial intelligence OR machine learning) AND cancer AND (imaging OR radiology OR radiotherapy OR computed tomography OR CT OR magnetic resonance imaging OR MRI OR ultrasound OR PET). Date query: July 9, 2024.

In oncology, there is an exponentially growing volume of research on AI, ML, and radiomics. Figure 1 shows that we had over 12 000 indexed articles in PubMed during 2023, with almost 60% of these related to either image analysis or radiomics for developing prognostic models.^4^

RO is particularly fertile ground for AI application, due to the daily use of radiological imaging (such as CT, MRI, and PET) and the preponderance of repetitive labour-intensive tasks such as tumour and organs at risk (OAR) delineation and radiation dose planning. Every patient encounter in RO generates vast amounts of quantitative data and qualitative images.^9^ These factors create an ideal environment for the clinically impactful use of AI.

**Figure 2.**
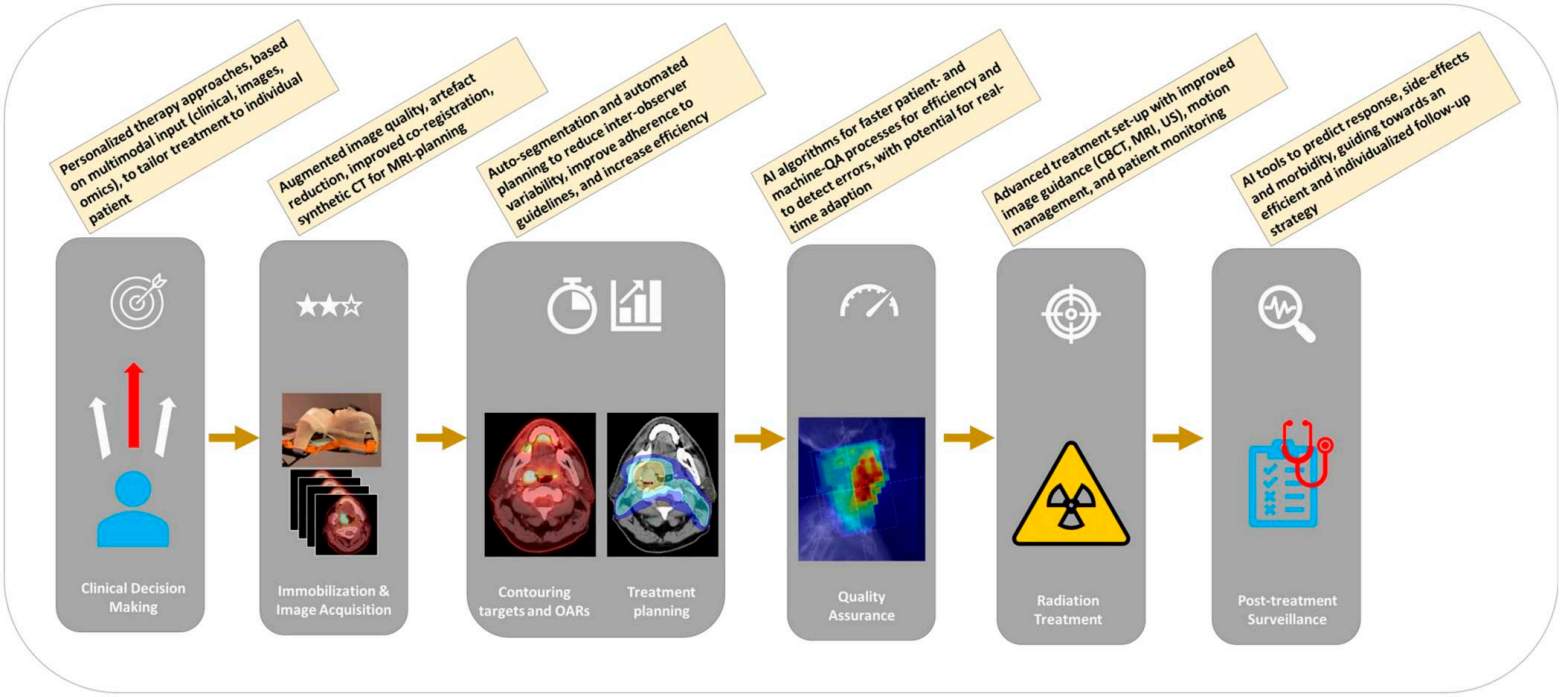
Radiation treatment workflow and anticipated AI-solutions within each step. AI = artificial intelligence.

Huynh et al.^10^ comprehensively explored the vast potential of AI across the entire RO workflow. This ranges from clinical decision support, for example, as employed in the SHIELD-RT trial,^11^^,^^12^ to process optimization (eg, immobilization, image acquisition,^13^ delineation of targets^14^ and OARs,^15^ and treatment planning,^16^ automated quality assurance of treatment delivery,^17^ and finally for response assessment^18^ and follow-up (see Figure 2). AI is thus potentially transformative at every step of a patient’s journey. See also the recent paper by Hurkmans et al.,^19^ which provides a guideline addressing the main topics regarding AI in RO.

## AI model development methodology: step by step

**Figure 3.**
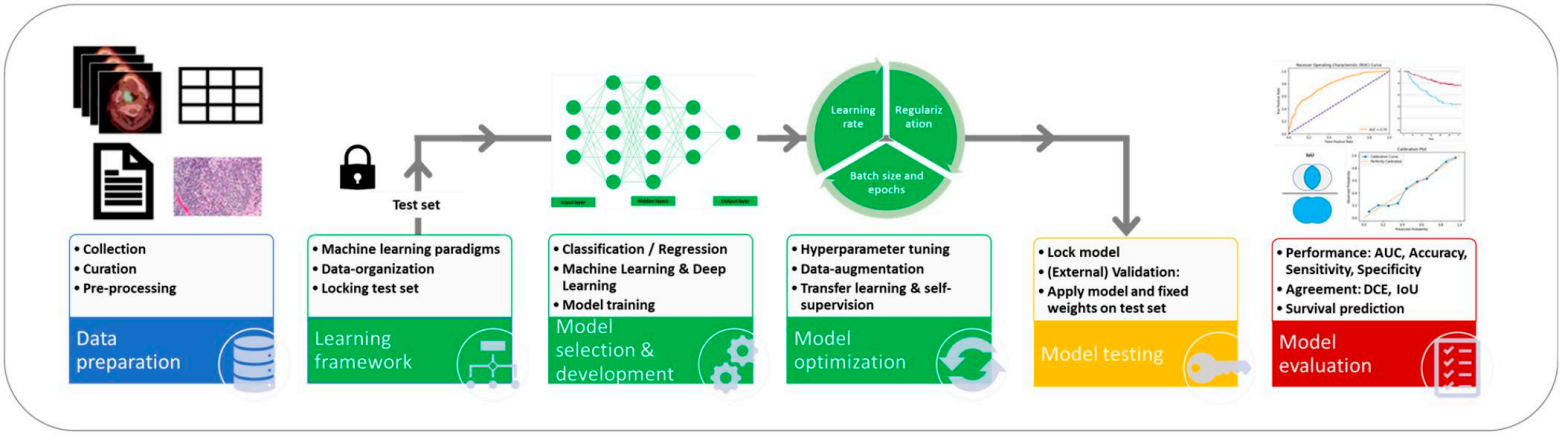
Schematic overview of steps in the process of the development of AI algorithm. AI = artificial intelligence.

Developing AI models follows a sequence of well-defined steps, and this can be easily visualized as a pipeline (see Figure 3). Adherence to these general principles increases the likelihood of producing a robustly generalizable and trustworthy AI for clinical use. A key step that might be overlooked in the heat of enthusiasm is to define an unambiguous clinical problem with an objectively measurable outcome. The clinical question becomes the moral compass that guides everything else that follows in model development and validation. Besides the nature of the training data and the technical output, an important consideration is the clinical task and its context in which the AI application is meant to work.

As an example, an AI supporting a treatment decision for a very expensive and potentially toxic drug will have different requirements in terms of performance and transparency than an AI supporting a decision to recommend a patient to exercise more. Also, the possibility to check and amend the results of an AI—as is the case, for instance, in tumour detection on images (auto-segmentation)—may change the requirements compared to an AI that makes autonomous decisions. Such requirements arising from the clinical task and context may influence the choice of ML algorithm, performance measures, and training/validation strategies. A generalized step-by-step approach will be described here.

### Data collection and preparation

This critical stage involves carefully specifying and extracting high-quality data relevant to the clinical problem. A detailed inclusion and exclusion criteria list is needed. In RO, the data typically needs to be extracted from institutional archives, such as Picture Archival and Communication System, treatment planning system, and electronic hospital records. This includes medical imaging, image annotations (ie, outlining anatomical regions of interest), and clinical case-mix variables (eg, diagnosis, staging, histopathological findings). Real-world RO data may be copious and available, but data quality assurance^20^ is required to address typographical errors, fill in missing values within the database (by going back to the original data source), remove duplicates, and adjust image annotations.

For images, RO adheres to the Digital Imaging and Communications in Medicine (DICOM) standard, which includes metadata that should be retained—vendor and model of scanner, image acquisition settings (eg, date and time of scan, field of view, direction of scan, current and voltage), and use of intravenous contrast. Image artefacts (eg, distortion, motion, scatter, noise) should be noted if these cannot be excluded from use. However, not all of the parameters of interest in RO will be recorded in the DICOM standard, so general information should be available separately from DICOM, such as the necessary immobilization/fixation devices, use of bolus materials, etc.

#### Data- and image-preprocessing

Pre-processing will depend on the design of the learning algorithm.^21^^,^^22^ Commonly, the scale and range of continuous variables need to be standardized prior to use in AI training; this is known as *normalization.* This prevents variables with larger magnitudes from overly dominating the model’s learning. This may include, for example, transforming the values to a distribution with mean 0 and SD 1, or clipping an image to a window of minimum and maximum intensity before scaling these to fall within the range 0-1. Other models may require the input image dimensions to be a fixed size, which involves cutting up a large image into smaller “patches,” or padding the voxels of small images with zero values around the edges. Various methods are available, each with pros and cons.^23^ Other pre-processing on images may include cropping to a specified dimension, or isotropical resampling of the image grid. Depending on the design, an algorithm may process images as 2D slices or as 3D volumes. Image analysis and normalization of CT scans is relatively straightforward and based on the Hounsfield Units calibration; however, for other image modalities in RO, including FDG-PET^24^ and MRI,^25^ this may be more challenging due to a lack of standardization and reproducibility.

#### Assessing fairness of data

An AI model only learns from the data it is presented with; therefore, assessing the representativeness of the data and adequacy of coverage of a given population is necessary.^26^ Biases in the data will likely lead to biased results, potentially disadvantaging certain patient groups.^27^ Selective inclusion may result in a loss of validity when the model is used in a different population.^28^ Researchers must carefully examine the training data to identify potential selection bias and confounding factors.^3^ We refer the reader to the paper by Cobanaj et al.^29^ for more details on this topic. Ideally, there should be no significant systematic differences in data availability, quality, annotation, and follow-up that might lead to disadvantage for a certain subgroup of patients.

### ML framework selection

**Figure 4.**
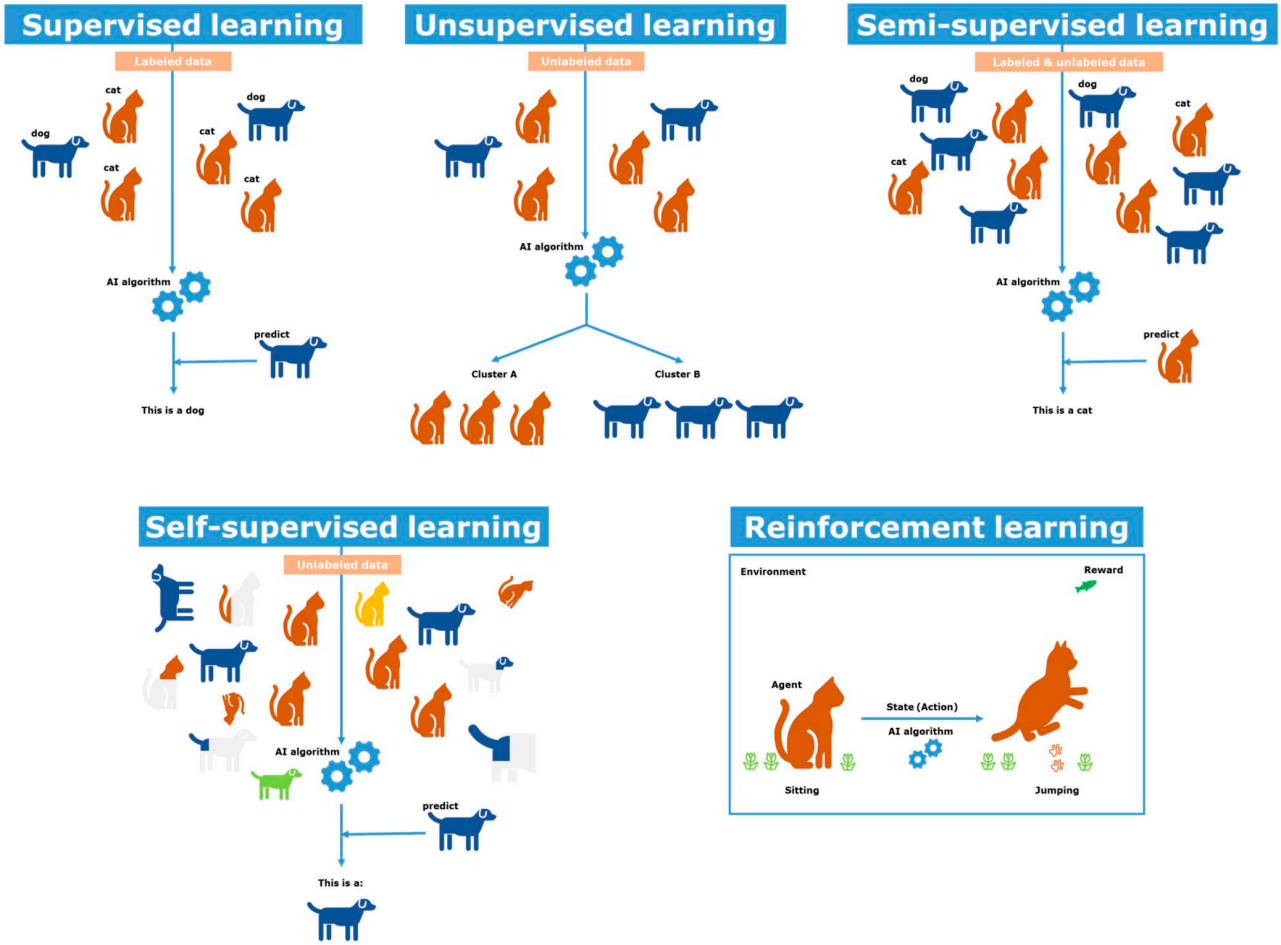
Machine-learning paradigms. In supervised learning, the model learns from (abstract) features and labels to predict classes. In unsupervised learning, the model learns to find patterns to cluster data. In this simplified example of cats and dogs, these patterns may be short vs long snout, pointy vs floppy ears, or long vs short tail. Semi-supervised learning combines both supervised and unsupervised learning using labelled and unlabelled data. Through the addition of unlabelled data, the performance can be improved without the need for additional expert-labelling. In self-supervised learning, the unlabelled data consists of both original data as well as processed data to augment the cohorts, with new images containing crops, or alterations in dimensions, in colour, or in orientation. In reinforcement learning, the agent learns to achieve a new state by trial and error receiving rewards and penalties. In this example, the cat (agent) learns to jump based on feedback after an action (rewards or penalties).

The type of ML framework used depends on the specific task and the type of available data (see Figure 4). Training paradigms are generally selected from the following options.

#### Machine-learning paradigms

During *supervised learning*, each item of input data used to generate the prediction must be exactly paired with an annotation (a.k.a. ground truth label) defining the desired result. For inference, only the input data (no annotation) are required, since a fully trained AI will try to mimic the desired response as output. Some common uses of supervised learning include diagnostic classification,^30^ outcome prognostication,^31^ object detection, and anatomical segmentation (delineation).^32^

*Unsupervised learning* differs fundamentally from supervised learning, since no annotation is needed. The AI model detects patterns, associations, and correlations within the training data without generating a prediction or a classification as a result. The most common outcome of such learning is to identify heuristic patterns, clusters, or sub-groups within the data, but without referencing any prior knowledge or clinical expertise^33^^,^^34^; this can be useful for clinical problems such as tumour subtyping using whole-genome assays.

One problem in the clinical domain is that suitable annotations and expertly generated ground truths might not exist or may prove too labour-intensive and time-consuming to obtain. To overcome this challenge, 2 approaches emerge. *Semi-supervised learning* applies 2 distinct learning submodules to a mixture of annotated and unannotated data; a “teacher” submodule employs supervised learning only on the labelled instances, while a “student” submodule trains with all the data, both labelled and unlabelled. The teacher’s predictions will constrain the student’s learning and thus provide a weak form of supervised learning to the student to ensure task relevance and minimize false associations. It is used in examples of disease classification^35^^,^^36^ and/or delineation.^37^

*Self-supervised learning* employs an architecture that self-generates surrogate labels to learn an implicit representation of the data; thus, it only needs unlabelled data. A commonly used “pretext” to train an implicit representation of the data is to intentionally blank out random portions of an input image, then train the model to fill in the missing portions. Once trained, the implicit representations stored in the AI memory can be transferred to a highly specialized downstream task, using only a small amount of labelled data in the process. Self-supervision is computationally intensive at the beginning, but downstream training is data-efficient and label-efficient; this could be of immense help in real clinical situations.

In contrast, *reinforcement learning* requires repeated interactions with an environment, for example, a human trainer. The AI model makes its best guess using what it has learned so far, and the trainer provides corrective feedback, which is used to update the model in memory. As long as this feedback loop is repeated many times, the model will keep refining its learning. This approach has been successfully employed for game-playing and self-driving vehicles but would also be highly applicable for clinical tasks such as radiotherapy treatment planning.^38^^,^^39^

#### Internal and external validation strategies

To obtain reliable (and hence generalizable) training and tuning/validation results, it is important to evaluate how the model might perform on an unseen dataset. The importance of validation is discussed in a later section. Partitioning and resampling strategies are especially important for smaller datasets. Guidelines, such as “Transparent Reporting of a Multivariable Prediction Model for Individual Prognosis or Diagnosis (TRIPOD),”^40^^,^^41^ provide a ranking based on the validation samples employed.

*Holdout* randomly assigns patients in the dataset into disjoint training and validation subsets; the held-out subset is only revealed at the very end, after the final model has been selected, serving as a final check on model performance. Some commonly used ratios for splitting are 80:20 or 75:25. However, holdout can introduce problems in small datasets because the sample size for training is reduced further, and the holdout subset may not be representative of the whole population.

*K-fold cross-validation* randomly divides the data into equally sized subsets (folds). The model is repeatedly trained and tested K times in total, using each fold 1 at a time for testing and the other (K-1) folds for training. The final model performance is an average result over K trials. This method is preferred for small datasets, as it may provide a more robust estimate of the model’s performance compared to holdout.

*Bootstrapping* creates new training sets of arbitrary size by randomly resampling patients from the original data, then replacing them back into the data (so it is possible that some patients might be redrawn multiple times), and repeating the process. This approach is also useful when the sample size is small or the underlying distribution is unknown. Similar to K-fold cross-validation, a model’s performance should be evaluated across multiple non-identical bootstrapped training sets. Steyerberg et al.^42^ provide a procedure for estimating model performance using bootstrapped training sets and then validating the model in the original (non-bootstrapped) dataset.

Stratification can be added to the above random resampling methods to ensure balanced class representation in training and validation sets. However, TRIPOD^40^ clearly distinguishes non-random resampling as a different class of modelling study (ie, type 2b and type 3) because random sampling likely produces validation data that are nominally identically distributed as the training data, but it might not be independent (eg, performed by unrelated clinicians, scanned with different devices). Hence, either non-random data partitioning (eg, patients enrolled in different studies or treated in non-overlapping calendar years) or using data from an entirely separate institution are considered a more independent evaluation of a model. Such notionally independent datasets may be called “independent test data” or simply “test data” in the field of AI to distinguish them from validation data as aforementioned. An important consideration regarding test data is whether it is both independent and identically distributed (henceforth “i.i.d.”) to obtain a precise estimation of final model performance.

### Model selection

#### Traditional models vs DL models

Traditional ML models work with structured data, where the essential variables and characteristics (“features” in AI parlance) have been engineered or extracted beforehand by human experts.^43^

**Figure 5.**
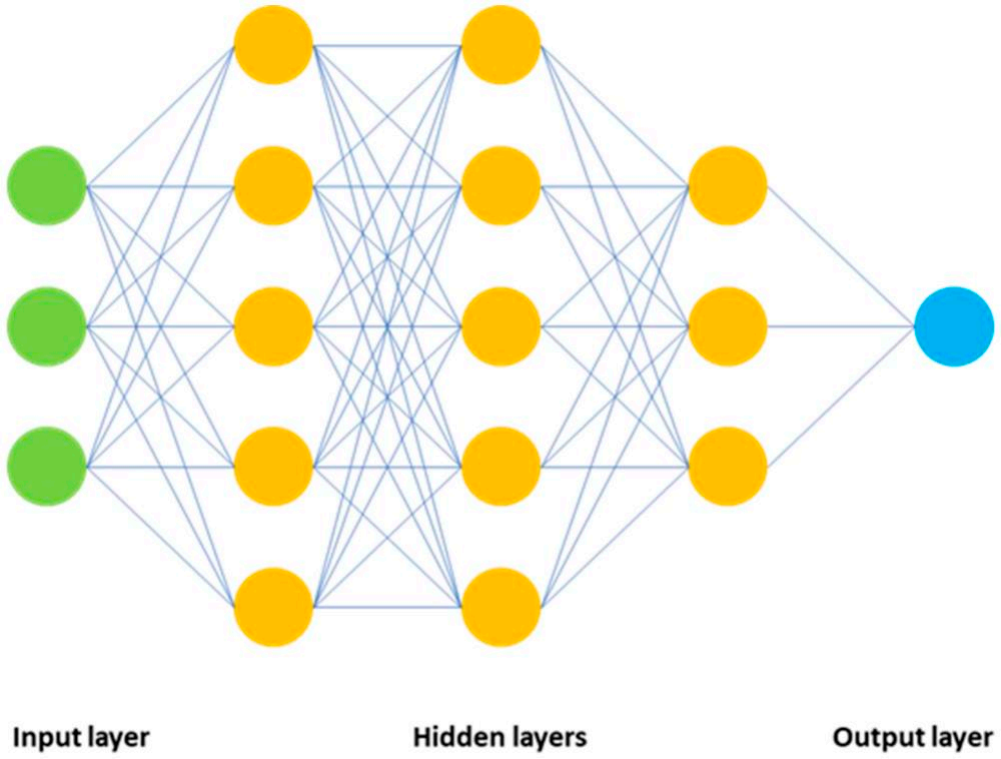
Artificial Neural Network (ANN) with input layer connected to several hidden layers, with multiple nodes and an output layer, which may provide a probability for a binary classification model.

Conversely, DL models do not require human intervention to engineer or define a feature space before training. As such, DL models self-construct a feature space that is required to execute a given task during their training cycles. An ANN is based on a conjecture about biological neural networks in the human brain. It consists of artificial neurons or “nodes” (see Figure 5) that are organized into layers, such that each layer receives input from the previous layer and passes its output to the next layer. Each node calculates the weighted average of all its inputs, then passes forward an output according to an activation function. A DL model is trained by optimizing the weights of the inputs to each node to minimize the error of its output relative to a known reference. “Deep” generally implies that there are 3 or more “hidden layers” of neurons sandwiched between an input layer and the final output layer.

As explained in Box 1, *radiomics* can either be based on traditional ML methods that analyse predefined image features relating, for example, the shape and the intensity of image pixels and their relative location in order to build a classification or prediction model from these radiomic features.^43^ This can be achieved by ML methods such as random forests, regularized regression, and a vast host of other ML models. Radiomics can also be based on DL methods, without the input of predefined features.

While traditional ML models offer a degree of interpretability, efficiency, and suitability with smaller datasets, DL models can achieve excellent results on par with some expert humans, but they lack the same degree of interpretability and transparency regarding their internal logic. A list of common models is given in Table 1.

**Table 1.** List of common prediction models.^a^

Model	Model description
*Traditional machine learning*	
Linear regression	This is a basic model architecture for predicting a continuous target variable. It works by fitting a straight line through the data points to make predictions.
Logistic regression	This is a model architecture used for classification problems that predicts the probability of an input belonging to a certain class, and then classifies the input based on a threshold value.
Survival models	Survival analysis models like the Cox proportional hazards model are trained using labelled data, where the time-to-event data (death, recurrence) and associated covariates are known. The model learns the relationship between the covariates and the survival outcome, allowing for predictions of survival probabilities or hazard ratios for new individuals
Decision trees	Decision trees are tree-like structures used for classification and regression, where the data are recursively split into smaller subsets, based on the values of the input features, and then making predictions based on the majority class or mean value in each subset.
Support Vector Machines (SVMs)	SVMs are employed in both classification and regression tasks by identifying a hyperplane that effectively separates data into distinct classes or predicts the value of a continuous target variable.
Random forests	Random forests are an ensemble learning method that combines multiple decision trees and is used mostly for classification and regression.
K-means	Unsupervised learning algorithms like k-means clustering are used to identify subgroups or clusters within the data to discover patterns in the data.
*Deep learning*	
Convolutional Neural Networks (CNNs)	CNNs are a type of artificial neural network (ANN) commonly used for image classification. See Box 2.
Recurrent Neural Networks (RNNs)	RNNs are a type of neural network commonly used for sequential data analysis, capturing information step-by-step and passing it into the next step, using recurrent connections and by creating memory. They are suitable for tasks involving temporal dependencies. In case of long-range dependencies, the RNN is vulnerable to maintaining information over long sequences, known as the “vanishing gradient” problem.
Transformers	Transformers are models that capture dependencies and relationships between different elements in a sequence within the data. For this, a self-attention mechanism is used to assess the importance of elements in the sequence. Unlike RNN’s, transformers process all data simultaneously in parallel, proving them more efficient in terms of computation and less prone to limitations in long-range dependencies.
Generative Adversarial Networks (GANs)	GANs are a type of DL model that aims to generate new synthetic data samples that are like a given training dataset. It applies a training process using a Generator that creates new sample data, which are iteratively evaluated by a Discriminator, until new realistic data have been generated that is indistinguishable from the real data.

a This is not meant as an exhaustive list of AI-related prediction models.

Abbreviation: DL = deep learning.

### Model development

After splitting the data into training and testing sets as described earlier, in the model development phase, the model is trained using a significant portion of the available data, and its performance is evaluated on a smaller part of the data for internal validation. If the model performs well during this evaluation, its parameters are frozen, and the model is locked. The model is then subjected to testing on an entirely new set of unseen data.

#### Model training

Once the data have been allocated into training, tuning/validation, and/or test sets, the development of the model may begin. Validation and test sets must not be exposed during model training to prevent an unrealistic estimation of model performance.^44^ It is crucial to note that when a model is assessed on the same data it was trained on, or on data that are randomly resampled from the same distribution as the training set, the estimate of model performance is at very high risk of appearing better than it truly is. This effect is known in the TRIPOD guidelines as “*over-optimism*.”

Training here refers to the iterative adjustment of the model’s internal parameters to minimize the difference between predicted and actual values. Convergence occurs when the model parameters change less than a small predefined limit, or when the difference between predicted and observed values consistently remains within a narrow range.

**Figure 6.**
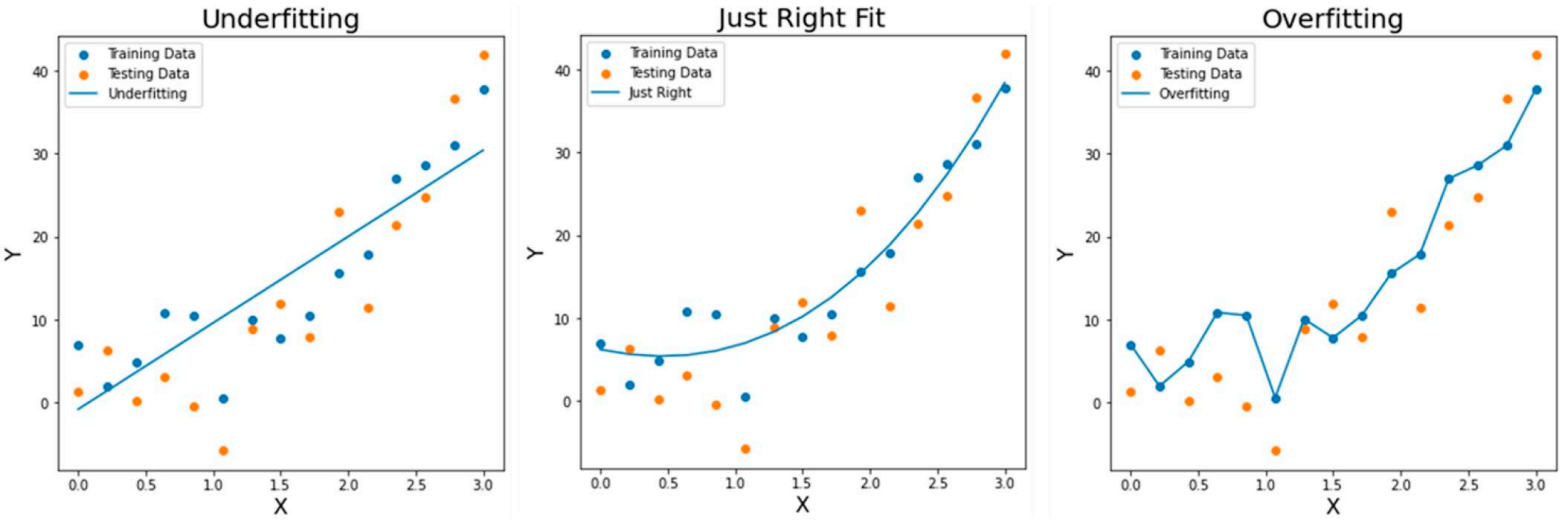
Model fitting of 2 variables (X and Y) based on input values of a training set (in blue dots). The model fit is provided as a solid blue line. The orange dots represent the test samples. In the left panel (“Underfitting”), it is evident that the model is not following the input data well (high bias), resulting in poor performance on unseen data. In the right panel (“Overfitting”), the model fit is too complex, following the training data too closely (high variance), again resulting in low performance on unseen data. The middle panel (“Just Right Fit”) has the optimal bias-variance trade-off which results in an improvement in performance on unseen data.

Common pitfalls during training include (1) overfitting—where the model starts fitting to random noise within the training dataset, thereby losing generalizability when evaluated against unseen data (ie, the validation and test sets), and (2) underfitting—where the model does not capture the true signal in the training dataset, thereby performing poorly in both training and validation/test datasets (see Figure 6).

Box 2. Convolutional Neural Networks (CNNs)

*Since CNNs are a cornerstone of DL in medical image analysis research, a brief explanation of their architecture is provided here. A CNN is designed to automatically identify certain patterns or features by training on large sets of images with associated labels. It consists of multiple interconnected layers, each responsible for performing a specific task in the image analysis process. The first layer is called the input layer, where the raw image data are fed into the network. There are several hidden layers, known as convolutional layers, that perform a series of mathematical operations called convolutions. These convolutions help the network to detect different features within the image, such as edges, textures, or shapes, at various levels of complexity. The convolutional layers are generally followed by pooling layers, which down-sample the feature maps obtained from the previous layers and reduce the spatial dimensions of the data while retaining the most important features. This process helps to simplify and compress the information for further processing. After several convolutional and pooling layers, the network typically has one or more fully connected layers, also known as dense layers. These layers analyse the extracted features and provide a probability for the presence of the label. In the training process, the network learns to recognize these patterns and features in the images by adjusting its internal parameters through a process called backpropagation, which “tunes” the weights of the previous layers. This training process allows the CNN to generalize its knowledge and make accurate predictions on new, unseen images.*

### Optimizing model performance

Training a model is an iterative process where we strive to find the right balance between high internal validity with good external generalizability. A modeler has some tools available that determine this balance.

#### Hyperparameter tuning

Hyperparameters are adjustable settings that control the learning process itself, as opposed to parameters that are learned directly from the training data. Therefore, hyperparameter tuning is used by the modeler to select the end state of a learning process and hence the model’s performance. Hyperparameters in ML models are highly model-specific. Commonly used settings in DL include:

1. *learning rate—*which determines the step size change of parameters during training. Larger step sizes allow parameters to converge rapidly but may also lead to uncontrolled feedback and unstable training;
2. *number of hidden layers—*which controls the extent of complexity that the model can capture. A network with more hidden layers is able to capture more complex relationships in the data; however, this also incurs more computation time;
3. *regularization—*which is a strategy to avoid overfitting and improve generalizability. One strategy involves adding lasso or ridge penalties, which, respectively, either incentivize model parameters to vanish (reduce to zero) or to be more uniform in value throughout. Another common method is dropout, which controls the rate at which randomly selected neurons in the hidden layer are directly set to zero. Dropout forces the network to learn across many neurons, rather than over-relying on a few neurons that might be picking out spurious information from the data;
4. *batch size—*which dictates how many training instances are processed together. Larger batch sizes are desired for faster training, but smaller batch sizes are needed when computer memory is limited;
5. *number of epochs—*which determines how many times the entire training set has been traversed. More epochs generally lead to better performance, but excessive training can lead to overfitting.

Some of the abovementioned settings overlap with ML models, such as regularization in penalized regression models (namely, ridge and LASSO penalties) and learning rate for gradient-boosted models.

#### Data augmentation

When dealing with limited datasets, augmentation artificially expands the training data by creating multiple random variations of existing instances. In cases of class imbalance issues, data augmentation can be used to create additional samples for the minority class, balancing the dataset and preventing bias towards the majority classes. For images, this may involve techniques such as cropping, flipping, rotation, scaling, adding noise, changing brightness or contrast, and applying geometric transformations.^45^ By learning from a broader range of examples that have been intentionally and randomly perturbed by differing amounts, the model is expected to become more robust and better at generalizing to unseen data, as demonstrated in a study on tumour target segmentation^46^ and the delineation of OAR for RO treatment planning.^47^

#### Transfer learning and foundation models

In certain situations with unusually limited training instances, more advanced techniques are needed. *Transfer learning* exploits pre-trained models developed for different but somewhat related tasks to the desired task. Pre-trained models are supposed to have learned generic features that can be repurposed for a new task.^48^ Thus, the pre-trained model is partially re-trained, usually by changing the learning rate in a few layers of the neural network while keeping all other layers fixed. The re-training is done in a supervised fashion with a limited training set for the new task. Results with ImageNet^49^ show that transfer learning can reuse features from natural photographs of everyday objects and successfully apply them to medical image analysis problems.

Fine-tuning of *foundational models* (FMs) is another possible approach when the training size is very small and reference annotations are rare. FMs are based on self-supervised models, which were discussed in an earlier section of this article. Note that, unlike models used in transfer learning, self-supervised models are not trained with any particular task in mind (ie, only a pretext task is used). Fine-tuning an existing FM requires much less data than training from scratch, and FMs are also vastly more adaptable to a wide variety of specialized tasks. Examples of this are the large natural language models (such as GPT and ClinicalBERT) that may be fine-tuned for diverse tasks ranging from chatbots for appointment bookings to obtaining structured summaries from electronic patient charts.

## Model evaluation metrics and interpretation

In previous sections, we have explained the importance of evaluating the final model’s performance on data that have not been exposed to training and optimization procedures. In this section, we focus instead on selecting the appropriate metrics for model performance and their correct interpretation. For a more detailed explanation, we refer to the work of Maier-Hein et al.^50^

### Classification

**Figure 7.**
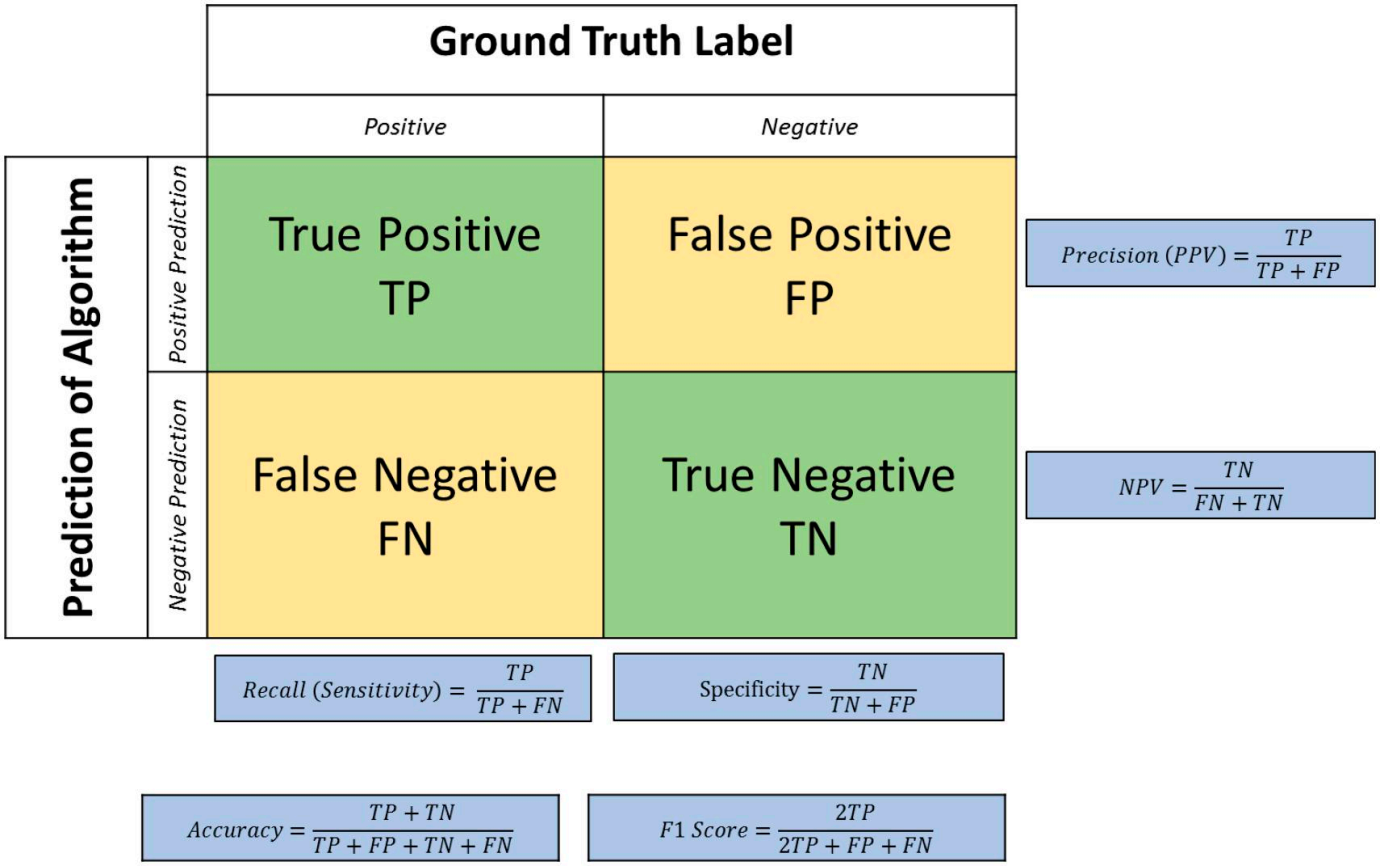
Contingency table, confusion matrix, for a binary classification algorithm, showing the distribution of predictions related to the ground truth labels with corresponding classifications. Cases that fall into the green cells are predicted accurately, cases in yellow falsely. Derived from this, several measures of performance can be calculated as indicated in the formulas presented in the blue boxes.

Classification models are evaluated based on the rates of true and false positives, as well as true and false negatives, derived from deviations from the ground truth labels. The implicit assumption is that the reference labels are perfectly correct, which may be problematic in tasks where even a human expert may be uncertain or at some risk of subjectivity. For dichotomous outcomes, the aforementioned true and false rates can be presented in a 2×2 table (also known as a *confusion matrix*). Various metrics can be computed from the table to describe a model’s discrimination performance (see Figure 7).

*Precision* is synonymous with Positive Predictive Value (PPV) and is the *ratio of true positives to all predicted positives*. In a clinical context, a model with high precision (ie, high PPV) would be useful if one wishes to select patients who would benefit from an expensive or potentially toxic treatment. Conversely, the Negative Predictive Value is the ratio of true negatives to all predicted negatives; this metric might be used to select patients who would not benefit from intensive, toxic treatment and may instead be offered treatment with fewer side effects.

*Recall* is synonymous with *Sensitivity—*this is the *rate of correct positive predictions relative to all positives*. Clinically, one would prefer a model with high recall (ie, high sensitivity) to avoid false negatives in scenarios such as cancer screening programmes. Conversely, Specificity is the rate of correct negative predictions relative to all true negatives. In the clinical setting, this is important to avoid treating healthy subjects or for the purposes of TNM staging or risk stratification for care planning.

The metric *Accuracy* is the proportion of all correct predictions over the entire population. This metric works accurately for balanced datasets (eg, where the numbers of responders and non-responders are roughly equal, or the ratio of diseased to healthy persons is close to 50:50). In highly unbalanced datasets, a prevalence-corrected accuracy should be reported. Alternatively, an *F1 score*, which is the harmonic mean of precision and recall, may be used.

The vast majority of models are probabilistic, meaning they do not predict a definitive outcome or classification; rather, the output is in the form of class probabilities. To use a model for decision-making, a decision threshold must be applied. This cut-off falls between 0 and 1 and defines the value that dichotomizes the probabilities as either one outcome or the other. Clinicians will recognize this decision threshold as the “operating point” on a receiver operating characteristic (ROC) curve. The locus of all values of Sensitivity and (1-Specificity) defines the ROC curve, thus the decision point (operating point) is the selection of a point along the ROC curve, typically as a balance between the desired sensitivity and specificity needed for a specific clinical decision. The model evaluation metric known as the Area under the ROC Curve (AUC-ROC) is used as a generalization of model discriminative ability across all possible decision thresholds. A model with an AUC-ROC of 1 is said to predict outcomes perfectly, whereas a model with an AUC-ROC of 0.5 is said to be no better than random chance, that is, it has the same success rate as if tossing a fair coin and calling out heads (or tails).

#### Discrimination vs calibration

While *discrimination* metrics assess how well a model differentiates between different classes of outcome, *calibration* instead assesses how well a model’s predicted probabilities match actual observed probabilities. It is entirely possible that a model may be highly discriminative but in fact poorly calibrated. A well-calibrated model provides accurate probabilities for the event of interest. Calibration can be inspected using graphical plots or using statistical tests, including, for example, the Hosmer–Lemeshow test^51^ as well as other measures.^52^

### Image segmentation models

Image segmentation is a subtype of classification problems where the classification into 1 or more regions of interest occurs on a voxel-by-voxel basis in a medical image. The most common application of this in radiotherapy is to delineate tumour volumes and organs-at-risk across multiple axial slices in a tomographic image. Voxel-wise metrics for evaluating AI model-generated delineations are directly analogous to the subject-level metrics defined above.^53^

In addition to the voxel-wise metrics, image segmentation evaluation also employs geometric overlap metrics between the output and the ground truth masks, such as the *Dice Similarity Coefficient* (DSC), *Intersection over Union* (IoU), and *Intraclass Correlation Coefficient* (ICC). These metrics quantify the area (in 2D) or volume (in 3D) that overlaps between the predicted and ground truth regions. Higher values of DSC, IoU, and ICC indicate better geometric agreement with the ground truth delineations.

**Figure 8.**
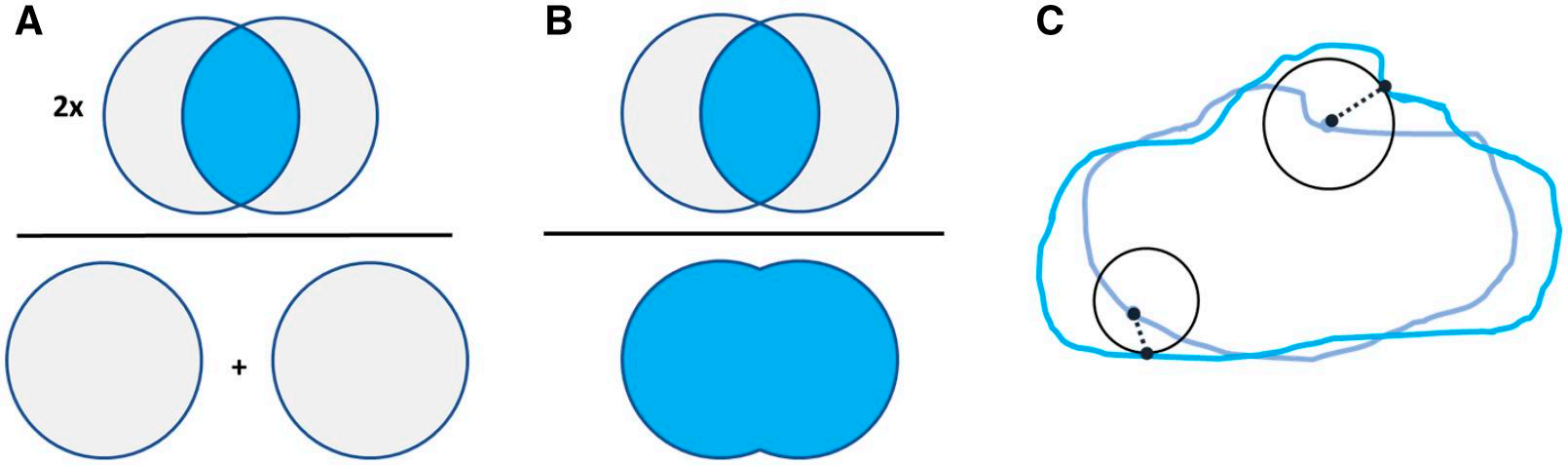
Graphical representation of common metrics describing geometric agreement. (A) Dice Similarity Coefficient (DSC) is defined as 2 times the intersection of 2 volumes divided by the total volume of both structures; (B) Intersection over Union (IoU) or Jaccard Index is the intersection divided by the union; (C): the Hausdorff Distance (HD) is measured as the maximum distance between 2 sets of points using the largest distance from a point in 1 structure to the closest point in the other one.

Furthermore, spatial agreement metrics, such as the *95th percentile Hausdorff Distance* and *average surface distance*, quantify how closely the predicted region boundary follows the ground truth boundary. It is important to note that lower values (smaller distances) indicate better agreement. Some examples are provided in Figure 8.

### Linear regression

A linear regression model predicts a continuously variable outcome; therefore, discrimination metrics are not suitable. Instead, the method of residuals is employed, which measures the distribution of the differences between predicted and observed values. Common metrics include Mean Squared Error, Root Mean Squared Error, Mean Absolute Error, Mean Percentage Error, and Mean Absolute Percentage Error. Lower values indicate a smaller difference between predicted and observed values, reflecting better model performance.

### Time-to-event models

The discrimination metric for time-to-event models, such as the Cox proportional hazards model, is the *Harrell Concordance Index*,^54^ commonly referred to as the concordance index or C-statistic. This method evaluates the ranked correlation of observed and predicted survival times, with a higher index indicating better predictive performance. Similar to classification tasks, survival analyses also require an assessment of calibration.^55^^,^^56^

## Challenges and opportunities in AI clinical translation

Despite the surge in AI publications in medicine, the implementation of these technologies has lagged behind. As of 2019, only a few dozen AI applications had received approval from the US Food and Drug Administration (FDA). However, this number has exceeded 880 as of May 2024, with 75% of these applications related to medical imaging alone.^57^ Several challenges must be addressed before AI can be more widely adopted.

### Clinical utility

**Figure 9.**
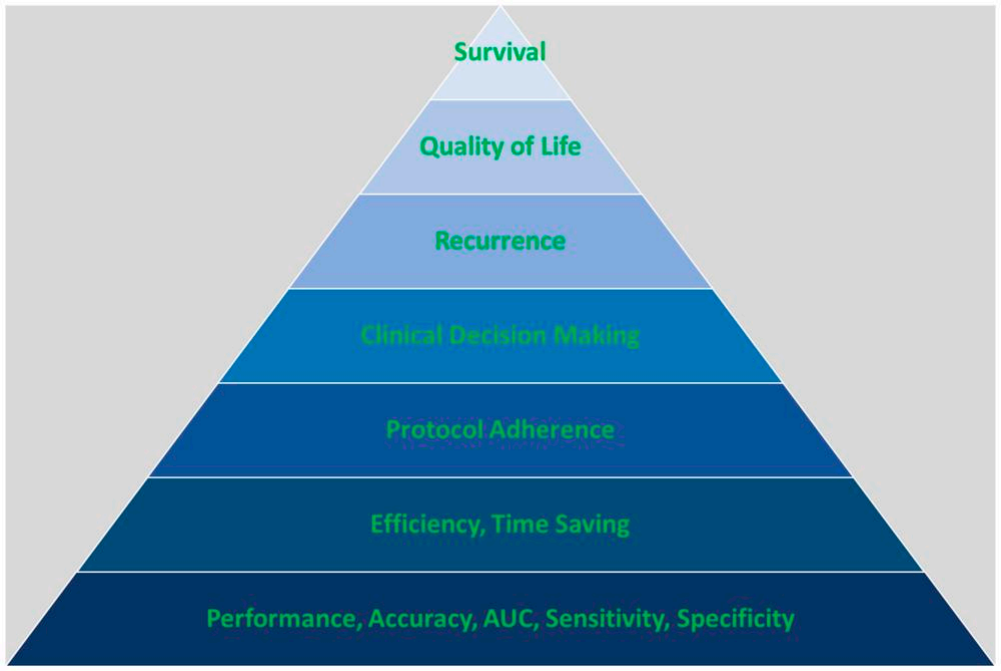
Pyramid describing different levels of clinical utility of an AI tool that incrementally improve on aspects in the clinical setting, moving away from performance measures only. AI = artificial intelligence

A highly accurate prediction model that has undergone the process of internal and external testing only does not meet the standards needed to apply the AI tool routinely.^58^ The ultimate question that needs to be answered from a clinical perspective is: “*What is the added value of the AI in clinical practice*?” Ultimately, the model will need to demonstrate clinical usefulness and improve on clinically relevant endpoints (see Figure 9). Depending on the type of application, endpoints that are relevant include *increased efficiency*, *reduced costs*, *changes in therapeutic approach*, *reduced toxicity* and *improved quality of life*, *improved control rates*, and *survival*. This gradual increase in clinical benefit for an individual patient is represented in the higher stages of the pyramid (Figure 9).

AI-powered automated organ segmentation tools can enhance *efficiency* and interobserver consistency when delineating tumours and OAR during radiotherapy planning.^14^^,^^15^^,^^59–63^ They also help improve *protocol adherence* for dose coverage and organ sparing.^16^^,^^64^ A personalized model-based approach may reduce futile treatments and enable more effective screening.^65^ AI-based *decision-making tools* have shown promise in randomized trials, such as SHIELD-RT, where an AI algorithm effectively stratified patients’ risk of needing emergency room visits during radiotherapy.^11^^,^^12^ Models predicting the risk of treatment side effects may guide treatment modality selection^66^^,^^67^; however, AI tools have yet to demonstrate a definitive improvement in clinically relevant outcomes, including *fewer recurrences*, improved *quality of life*, and increased *survival*. In general, there is a lack of studies demonstrating added clinical value for implementation of AI tools.^68^

Future research must focus on developing AI models that will lead to long-term changes in patient outcomes. One such example is an AI model that predicts which patients will benefit from androgen deprivation therapy when administered alongside radiation therapy in prostate cancer.^69^ This model is currently available for clinical use in the United States.

Overall, AI continues to hold great promise for improving efficiency and enhancing decision-making in medicine, but wider adoption will depend on demonstrating clear and tangible benefits to patients.

### Reproducibility and transparency

To bridge the gap between research and real-world application, improving the reproducibility of AI research findings is essential. This requires enhanced study design, reporting, and data-sharing protocols. Data should adhere to the principles of Findable, Accessible, Interoperable, and Reusable (FAIR)^70^ to promote transparency and testability. General model reporting guidelines, such as TRIPOD,^40^ should be adopted, along with additional considerations for developing and evaluating AI models, like TRIPOD-AI,^41^ which was recently released and now supersedes TRIPOD-2015^71^ and focuses on ensuring transparency in reporting for AI in healthcare,^72^ while the CheckList for EvaluAtion of Radiomics research (CLEAR) pertains to AI models in medical imaging providing the minimum requirements for presenting clinical radiomics research.^73^^,^^74^^,^^75^ Open-source data repositories, including The Cancer Imaging Archive (TCIA)^76^ and the NCI Imaging Data Commons,^77^ are essential resources for clinical AI development. Additionally, repositories of AI models, such as modelhub.ai, and model registries, such as AIMEbio, support open access to trained models for new research and for studies aimed at reproducibility and generalizability.

### Generalizability

The generalizability of AI models remains a significant concern,^78^ as sampled data may not accurately reflect the true diversity of real-world patients. Access to multi-institutional data from various contexts can help mitigate the under-representation of subgroups based on practice setting (academic vs community), ethnicity, geographic location (which influences cancer aetiology), and institutional protocols. However, it is crucial to recognize that models might learn from site-specific artefacts present in scans rather than from clinical indicators.^79^

When deploying AI models, transparency regarding the training and testing data is essential. A model’s application should be limited to populations that are similar to those included in the training dataset.^78^ Additional preclinical testing, both retrospective and prospective, within the intended practice setting is recommended. Randomized trials may be necessary to confirm the superiority of an AI tool, particularly if it suggests changes to patients’ care pathways. Phase 3 trials in medicine are rare,^80–83^ and currently, none have been reported in RO.

“*Continuous learning*” AI is an emerging concept that moves away from static models trained on fixed datasets. These models are designed to autonomously update themselves with new data over time. An example of this is provided in the paper by Morin et al.,^84^ in which a learning infrastructure is developed using multimodal health data that are systematically organized and assessed for data quality from large populations of patients with cancer, each with numerous datapoints, intended to apply AI for individual prognosis. This approach has been proposed to counteract the gradual performance degradation of a model over time due to changes in clinical practice.^85^ However, it will require routine monitoring to detect any unanticipated effects that may arise as the model is continuously updated.

### Explainability and trust

AI models in medicine are often referred to as “*black boxes*,” reflecting the difficulty for doctors to understand the reasoning behind model predictions. This lack of interpretability leads to decreased trust and acceptance by clinicians,^86^ resulting in a biased preference for human judgement (“*algorithm aversion*”), even when an AI can be objectively shown to outperform human experts.^87^^,^^88^ To mitigate the black box effect, researchers have proposed “*explainable AI*” methods, including the use of saliency maps, which graphically represent which parts of an image are most important to the model’s prediction^89^ (eg, using Gradient-weighted Class Activation Mapping, or Grad-CAM). However, it must be recognized that the trustworthiness of saliency maps for abnormality localization may be limited,^90^ and these maps should be viewed merely as a sanity check to confirm the location.

Lastly, healthcare providers’ trust in AI is influenced by their understanding of the model development process, including its vulnerabilities, pitfalls, and strategies. Future medical education needs to incorporate digital literacy training on AI to bridge this knowledge gap.^91^ Peer-to-peer education from clinician-informaticians holds promise for increasing knowledge, acceptance, and confidence in AI.^92^

## Role of the clinician

To improve the adoption of AI, a key factor for success is “*human–machine teaming*” to foster trust and support from clinicians.^93^ This concept, also referred to as “*physician-in-the-loop*,” emphasizes clinical collaboration throughout the AI model development process. Clinicians serve as authorities, providing feedback on various aspects of the research question. They should contribute their expertize in areas such as:

1. *Clinical problem definition—*clarifying the intended goal of the model.
2. *Data verification—*ensuring that the data and annotations are of sufficient accuracy for model training.
3. *Model evaluation—*reviewing and assessing the model’s outputs (eg, segmentation regions) to identify potential issues and unexpected consequences.
4. *Understanding underperformance—*analysing and diagnosing model underperformance in collaboration with data scientists to pinpoint sources of error.
5. *User interface—*working closely with medical physicists and/or vendors to help design user-friendly interfaces for AI tools, ensuring that these tools are intuitive and can be seamlessly integrated into clinical workflows.

This collaborative paradigm should not only benefit AI development but also build trust among the involved physicians.

## Recent developments and future perspectives

The field of AI is evolving rapidly, with new developments being reported that have the potential to further enhance applications in medicine.

*Multimodal models* incorporate different types of medical data, including imaging, pathology, genetics, and data from electronic health records such as medical history, physical examination, vital signs, and laboratory data, as well as information from wearables.^94^ This approach may pave the way for personalized medicine in oncology.^95^

*Foundation models* are basic systems with broad capabilities, trained on large datasets to establish strong baseline performance. Other, more specific AI models can be built on this foundation. Unlike many AI systems that are trained and deployed for specific tasks, foundation models are designed to be adaptable frameworks that can be applied to a wide range of purposes.

Significant attention has also been given to *generative AI*, which refers to a type of AI that generates new content, such as images or text, based on specific inputs (prompts). This is achieved by training a model to learn patterns and characteristics from existing data and then generating new data based on that knowledge. Examples in the public domain include large language models (LLMs), such as ChatGPT (Chat Generative Pre-trained Transformer), and models that create images from text inputs. LLMs serve as examples of foundation models, as described earlier. In the medical field, generative AI may be able to produce medical images, assisting in data augmentation for training. Generative models may also have the potential to generate medical reports,^96^ read and interpret clinical notes,^97^ create patient summaries, or assist in responding to patient questions,^98^ although additional research is needed before these models are ready for clinical deployment.^99^

The concept of *federated learning* enables the training of algorithms across multiple decentralized devices or servers holding local data samples without exchanging their data. This approach balances the need for advanced ML with the imperative of data privacy and security.^100^^,^^101^

The technical advances in medical AI have the potential to transform healthcare and improve patient outcomes in the future. The role of healthcare professionals in RO may shift, allowing them to spend less time on routine tasks and more time with patients.^10^ In this rapidly changing field, regulatory bodies must establish appropriate legal and ethical guidance to promote the safe use of AI and foster its application.^102^

## Conclusions

AI in RO holds promise with many proposed applications, particularly in improving efficiency through techniques such as auto-segmentation and auto-planning. However, the clinical use of prediction models that impact patient decision-making remains limited. Training physicians in AI applications is essential to enhance multidisciplinary collaboration between data scientists and clinicians, thereby fostering acceptance and trust in these technologies.
